# Particle-attached Microbes in Eelgrass Vegetation Areas Differ in Community Structure Depending on the Distance from the Eelgrass Bed

**DOI:** 10.1264/jsme2.ME23013

**Published:** 2023-09-02

**Authors:** Md Mehedi Iqbal, Masahiko Nishimura, Masayoshi Sano, Susumu Yoshizawa

**Affiliations:** 1 Atmosphere and Ocean Research Institute, The University of Tokyo, 5–1–5 Kashiwanoha, Kashiwa, Chiba 277–8564, Japan; 2 Department of Natural Environmental Studies, Graduate School of Frontier Sciences, The University of Tokyo, 5–1–5 Kashiwanoha, Kashiwa, Chiba 277–8563, Japan

**Keywords:** *Zostera marina* (eelgrass), 16S rRNA, particle-attached and free-living bacteria, particle formation, organic matter

## Abstract

*Zostera marina* (eelgrass) is a submerged flowering plant often found in the coastal areas of Japan. Large amounts of suspended particles form in highly productive environments, such as eelgrass beds, and the behavior of these particles is expected to affect the surrounding microbial community. We investigated the microbial community structure of suspended particles in three eelgrass fields (Ikuno-Shima Is., Mutsu Bay, and Nanao Bay) and inferred the formation and dynamics of suspended particles from a microbial community structure ana­lysis. Seawater samples were collected directly above each eelgrass bed (eelgrass-covering) and from locations dozens of meters away from the eelgrass bed (bare-ground). In consideration of the two different lifestyles of marine microbes, microbial communities were obtained from particle-attached (PA) and free-living (FL) states. Differences in microbial diversity and community structures were observed between PA and FL in all eelgrass beds. The FL microbial community was similar between the two sampling points (eelgrass-covering and bare-ground), whereas a significant difference was noted in the microbial community structure of suspended particles between the two sampling points. This difference appeared to be due to the supply of organic matter from the eelgrass sea ground and leaf-attached detritus produced by microbial activity. In addition, the classes *Flavobacteriia*, *Alphaproteobacteria*, and *Gammaproteobacteria* were abundant in the PA and FL fractions. Furthermore, many sequences of the key groups (*e.g.*, *Planctomycetes* and *Verrucomicrobia*) were exclusively detected in the PA fraction, in which they may circulate nutrients. The present results provide insights into the microbial communities of suspended particles and provide the first step towards understanding their biogeochemical impact on the eelgrass bed.

Seagrass beds are of great importance as a source of organic matter for marine life, particularly heterotrophic bacteria, and protists (Holmer *et al.*, 2004; Fraser *et al.*, 2016). Marine organic matter is roughly classified into particulate organic matter (POM) and dissolved organic matter (DOM) depending on its form of existence. POM forms in sea areas with high primary production, whereas decomposition activity by microorganisms is also active in these areas. Seagrass plants have been shown to trap and sink suspended POM originating from both external sources and leaf shedding inside the seagrass bed (Terrados and Duarte, 2000; Vonk *et al.*, 2008; Gillis *et al.*, 2014). Once suspended POM is deposited within seagrass beds, the seagrass canopy inhibits the matter from being resuspended, and consequently, most POM recycles within the bed by microbial activity, releasing nutrients to the surrounding environment (Peduzzi and Herndl, 1991; Gillis *et al.*, 2014). Although microbe-particle interactions are essential for the degradation of organic matter and regeneration of nutrients, they have not yet been examined in detail in seagrass beds.

Marine particles are colonized and decomposed by particle-attached (PA) microorganisms, releasing DOM into the surrounding water layer, fueling free-living (FL) microorganisms (Kiørboe and Jackson, 2001; Azam and Malfatti, 2007; Benner and Amon, 2015; Li *et al.*, 2021). Based on differences in the quality of organic matter available to marine microbes, the microbial community structure may differ between PA and FL microbial communities (DeLong *et al.*, 1993). Previous studies demonstrated that the PA fraction was relatively enriched by polymer-degrading bacteria (*i.e.*, *Flavobacteria*, *Gammaproteobacteria*, *Planctomycetes*, *Firmicutes*, and *Verrucomicrobia*), whereas the FL fraction was enriched by members of *Alphaproteobacteria* (Crespo *et al.*, 2013; Bižić-Ionescu *et al.*, 2015; Milici *et al.*, 2017; Li *et al.*, 2021). However, several research groups reported a similar microbial community structure between the PA and FL fractions (Hollibaugh *et al.*, 2000; Ghiglione *et al.*, 2007; Ortega-Retuerta *et al.*, 2013). Some of the major microbial groups, such as *Alphaproteobacteria*, *Gammaproteobacteria*, and *Bacteroidetes*, have the ability to switch their lifestyles between PA and FL (Yung *et al.*, 2016). Other studies investigated PA and FL microbial communities in various marine environments (Crespo *et al.*, 2013; Mohit *et al.*, 2014; Rieck *et al.*, 2015; Salazar *et al.*, 2015; Tarn *et al.*, 2016; Milici *et al.*, 2017; Suzuki *et al.*, 2017; Li *et al.*, 2021). However, there is currently no information on the PA and FL fractions in eelgrass beds, particularly *Zostera marina*, a representative species of Japanese eelgrass.

*Z. marina* (eelgrass) is a widely distributed seagrass species that generates ecological diversity and economically important ecosystems along coastlines throughout much of the Northern Hemisphere (Marba *et al.*, 2006; Duffy *et al.*, 2015; Fahimipour *et al.*, 2017). In eelgrass beds, the PA microbial community changes its niche from the particle surface to a solid surface (*e.g.*, eelgrass leaves) through particle migration and attachment processes, while suspended particle microbes were also detected in a limited eelgrass area (Iqbal *et al.*, 2023). Therefore, further studies are needed on the particle formation process over a wide spatial range to obtain a more detailed understanding of the relationships between suspended particles and eelgrass-inhabiting microbes.

Microbial 16S rRNA gene amplicon sequencing has been used to clarify the microbial diversity of eelgrass samples in our series of studies. As the first step to clarify the microbial consortia, we reported the microbial community structure in an eelgrass bed growing at Futtsu clam-digging beach, Tokyo Bay (Iqbal *et al.*, 2021). We recently described the role of marine particles during the eelgrass growing season (Iqbal *et al.*, 2023). However, these studies were conducted on limited spatial scales. Therefore, the present study investigated the formation process of suspended particles in eelgrass beds from 16S rRNA sequence data using various eelgrass samples collected from three eelgrass fields in Japan: Ikuno-Shima Is., Mutsu Bay, and Nanao Bay. This is important because suspended particles act as a means of transportation for microorganisms between seagrass leaves and the surrounding seawater (Iqbal *et al.*, 2023), and PA microbes play an important role in bio-element cycling (Bachmann *et al.*, 2018). By comprehensively characterizing the microbial dynamics of suspended particles, the present study provides important insights into the role of particles in eelgrass beds and represents the first step towards obtaining a more detailed understanding of the biogeochemical impact of PA microbes in the seagrass-based ecosystem.

## Materials and Methods

### Site description and sample collection

**Fig. 1.**
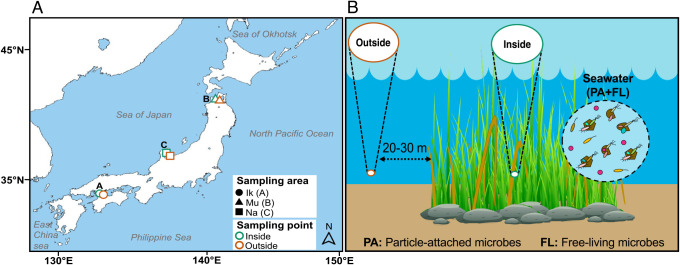
Sampling locations and strategy. (A) Samples were collected from three *Zostera marina* eelgrass fields (Ik: Ikuno-Shima Is.; Mu: Mutsu Bay; Na: Nanao Bay) in Japan. (B) At each sampling area, samples were collected from the inside (eelgrass-covering) and outside (bare-ground) of the eelgrass bed.

Seawater samples were collected from three *Z. marina* eelgrass fields (Ikuno-Shima Is., Mutsu Bay, and Nanao Bay; Fig. 1A) in summer (June-August 2015) during low tide (water depth of 0.8–1.4 m; Table 1).

Sampling details are shown in Table 1. The Ikuno-Shima eelgrass area (34°17′52.92″N, 132°55′02.91″E) is in Hiroshima Prefecture in western Japan’s mainland. This eelgrass area is very calm and quiet, the bottom of which is covered with fluffy mud. The Mutsu Bay eelgrass area (40°51′58.06″N, 140°50′00.04″E) is in Aomori Prefecture, the northernmost prefecture of Japan’s mainland. The Nanao Bay eelgrass area (37°05′10.21″N, 136°52′14.62″E) is in Ishikawa Prefecture facing the Japan Sea, with a weak tidal stream.

Test samples used for a microbiome ana­lysis and environmental measurements were simultaneously collected. Temperature and salinity were measured using COMPACT-CTD (ACTD-CMP; JFE Advantech), while pH and oxidation-reduction potential (ORP) were measured using a portable multi-meter (DM-32P; DKK-TOA CORPORATION). All environmental measurements were obtained at a depth of 50‍ ‍cm. In a microbial ana­lysis, seawater samples were collected in triplicate on the inside (eelgrass-covering) and in duplicate on the outside (bare-ground: 20–30‍ ‍m from the edge of the eelgrass patch) of the eelgrass bed (Fig. 1B). A water sample was collected in a sterile plastic bottle (1 L) using a gloved hand from the surface (0.1–0.5 m) layer, filtered with a 3.0-μm pore-size cellulose acetate membrane filter (ADVANTEC), and then filtered again with a 0.2-μm pore-size membrane filter (ADVANTEC) to collect PA and FL microbes, respectively (Iqbal *et al.*, 2023). All filters were immediately stored at –80°C until further ana­lyses.

### DNA extraction, PCR amplification, sequencing, and raw data treatment

DNA was extracted in triplicate for inside samples and in duplicate for outside samples, except for FL inside samples (*n*=2) collected from Ikuno-Shima Is. DNA extraction was performed using a DNeasy PowerSoil DNA extraction kit (Qiagen) according to the manufacturer’s instructions with slight modifications as described in a previous study (Iqbal *et al.*, 2023). Briefly, for DNA extraction from suspended particles, a 3.0-μm pore-size filter with trapped particles was cut into small pieces and placed directly into an extraction tube. To extract DNA from the FL fraction, a 0.2-μm pore-size filter with trapped FL microbes was cut into small pieces and placed directly into another extraction tube in the same manner. Extracted DNA was cleaned with a NucleoSpin gDNA Clean-up kit (MACHEREY-NAGEL GmbH & Co. KG) according to the manufacturer’s instructions and stored at –30°C until further use.

The hypervariable V4 region of the 16S rRNA gene was amplified in PCR reaction liquid using the primers 515F (5′-GTGCCAGCMGCCGCGGTAA-3′) and 806R (5′-GGACTACHVGGGTWTCTAAT-3′: Caporaso *et al.*, 2012). V4 regions were chosen for sequencing because they are capable of detecting both bacterial and archaea taxons with high resolution and show few biases (Wear *et al.*, 2018). PCR was performed under previously described conditions (Iqbal *et al.*, 2023). Three technical PCR replicates were performed for each sample, pooled, purified, and paired-end sequenced (2×300) on the Illumina MiSeq platform (Illumina).

Raw reads were processed and analyzed using the program Quantitative Insights Into Microbial Ecology 2 (QIIME2) version 2020.2 (https://qiime2.org/). Raw paired-end FASTQ reads were demultiplexed using the Fastq barcode splitter (http://hannonlab.cshl.edu/fastx_toolkit/index.html) and imported into QIIME 2. Demultiplexed sequences were quality filtered, denoised, chimera checked, and dereplicated using a DADA2 denoise-paired plugin (Callahan *et al.*, 2016) after an inspection of the quality profile plots of forward and reverse reads. Following the truncation of the 270-bp forward reads and 250-bp reverse reads with a high quality score (the lower whisker was 25). The taxonomic affiliation was performed using q2-feature-classifier classify-sklearn on the Greengenes (13_8 release) reference database (McDonald *et al.*, 2012). Alignment was conducted with the Mafft algorithm (Katoh and Standley, 2013) to build a phylogenetic tree using the Fasttree software (Price *et al.*, 2009) for its subsequent use in the UniFrac (Lozupone *et al.*, 2006) distance ana­lysis. The amplicon sequence variant (ASV) table resulting from DADA2 was filtered to exclude sequences classified to organisms other than Bacteria and Archaea (*i.e.*, eukaryote, chloroplast, mitochondrial, and unclassified sequences). Sequences were rarefied at the lowest number of reads (29,885) obtained within all the samples to reduce any bias caused by a difference in the sequencing depth during diversity estimations.

### Data visualization and statistical ana­lyses

Microbial community composition and diversity ana­lyses were performed by processing the ASV table in the R environment (R Core Team, 2020) with the Phyloseq (McMurdie and Holmes, 2013), Microbiome (Lahti and Shetty, 2012–2019), Vegan (Oksanen *et al.*, 2020), and ggplot2 (Wickham, 2016) packages.

Richness estimators (Chao1; Chao *et al.*, 2005), diversity indices (Shannon; Shannon and Weaver, 1949), and observed ASVs (species) were calculated in R software. To compare the microbial communities (*i.e.*, beta diversity) associated with the PA and FL fractions among sampling types, namely, areas (Ikuno-Shima Is., Mutsu Bay, and Nanao Bay) and points (inside and outside), we measured unweighted UniFrac (Lozupone and Knight, 2005; Lozupone *et al.*, 2006) dissimilarities in R using the Phyloseq package. These dissimilarities were then plotted using a principal coordinate ana­lysis (PCoA) and non-metric multidimensional scaling (NMDS). Regarding both two fractions (PA and FL), a two-way PERMANOVA test was performed via the “adonis” function of “Vegan” packages (Oksanen *et al.*, 2020) with 9,999 permutations to assess the significance of differences in community compositions among the sampling types: areas (Ikuno-Shima Is., Mutsu Bay, and Nanao Bay) and points (inside and outside). A one-way PERMANOVA test was also applied for eelgrass-covering waters (inside) to identify differences between lifestyles (PA and FL). In addition, to detect taxa with a significant differential abundance between the PA and FL fractions of eelgrass-covering waters (inside), a linear discriminant ana­lysis effect size (LEfSe) measurement (Segata *et al.*, 2011) was created according to the web-based tool (Chong *et al.*, 2020) as previously reported (Iqbal *et al.*, 2023).

The Kruskal-Wallis test was performed in R to assess the significance of differences among groups for alpha diversity matrices (Chao 1, Shannon, and Observed ASVs), and the Wilcoxon rank-sum test with a false discovery rate (FDR) correction was then applied to measure pairwise group differences. In addition, a one-way ana­lysis of variance (ANOVA) of eelgrass-covering waters (inside) was performed to examine the significance of differences in major microbial taxa between lifestyles (PA and FL). Before the statistical ana­lysis, the distribution of each variable was checked for normality and the homogeneity of variance using the Shapiro-Wilk test.

### Sequence data accession number

Sequence data have been deposited in the DNA Data Bank of Japan (DDBJ) Sequence Read Archive (DRA) under the accession number DRA014823.

## Results and Discussion

The present study originally investigated whether specific changes in microbial communities exist among eelgrass beds over a wide geographical area in Japan (Ikuno-Shima Is., Mutsu Bay, and Nanao Bay) by focusing on microbial lifestyles: the PA and FL states, and water-sampling points: eelgrass-covering (inside) water and bare-ground (outside) water. 16S rRNA gene amplicon sequencing was used to characterize the bacterial and archaeal microbial communities of eelgrass samples. This approach differs from previous studies in that we focused not only on a geographical location and spatial difference, but also on two microbial lifestyles: the PA and FL states.

Environmental data from three sampling areas (Ikuno-Shima Is., Mutsu Bay, and Nanao Bay) with two sampling points (inside and outside) are shown in Table 1. Temperature, pH, and salinity were similar among the sampling points, but fluctuated among the sampling areas. Salinity varied from 30.6 to 32.7 among the sampling areas, with the highest salinity being observed in Mutsu Bay and the lowest in Nanao Bay. Temperature varied between the maximum (28°C) and minimum (19°C) values, with the highest temperature being observed in Nanao Bay and the lowest in Ikuno-Shima. pH values were similar at all sampling points (Table 1). However, ORP values fluctuated with changes in both the sampling points and areas (Table 1).

By targeting the V4 hypervariable region of 16S rRNA, 3,036,881 paired-end raw reads were obtained using the Illumina Miseq platform. After quality-filtering and discarding chimera, chloroplast, mitochondrial, and unassigned sequences, 2,117,189 high-quality sequences were retained for ana­lysis (Table S1). The average number of reads was 73,006 per sample (min=29,885; max=110,087) and 6,267 ASVs were obtained. In the estimation of diversity, the number of sequences per sample was rarefied at 29,885 sequence reads (Table S1). Rarefaction curves were obtained with the number of normalized ASVs, and the Shannon alpha diversity index nearly reached the saturation level for all samples (Fig. S1).

**Fig. 2.**
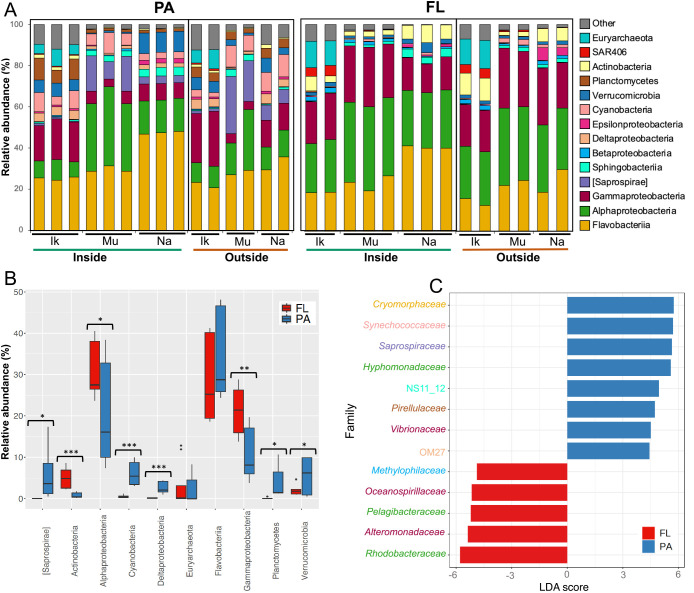
Significant differences in the microbial community composition among sample types. (A) Microbial community compositions of the particle-attached (PA) and free-living (FL) fractions of eelgrass-covering water (inside) and bare-ground water (outside) collected from three *Zostera marina* eelgrass beds (Ik: Ikuno-shima Is.; Mu: Mutsu Bay; Na: Nanao Bay) at the phylum level. Two phyla, *Proteobacteria* and *Bacteroidetes*, are shown at the class level. The groups under 2% were combined and called “Others”. (B) Comparisons of the top 10 microbial groups between the PA and FL fractions of eelgrass-covering water (inside). Asterisks indicate significant differences (ANOVA, *P<0.05, **P<0.01, ***P<0.001). (C) Linear discriminant ana­lysis (LDA) effect size (LEfSe) comparison of differentially abundant taxa between the PA and FL fractions (only eelgrass-covering water samples) at the family level. Each family was color-coded according to the taxonomic phylum/class. Differences were significant (*P*<0.05; Kruskal–Wallis test) between groups (PA vs FL). The threshold of the logarithmic LDA score was >3.0. A negative LDA score represents depletion in PA and enrichment in FL (red), while a positive LDA score represents the opposite (blue).

The taxonomic ana­lysis of sequencing data revealed differences in microbial compositions among sample types: microbial lifestyles (PA and FL), sampling points (inside and outside), and sampling areas (the domestic eelgrass beds investigated herein). The relative abundance of major microbial groups is shown at the phylum/class level in Fig. 2A and 2B. Regarding eelgrass-covering waters (inside), a LEfSe ana­lysis was used to characterize microbial communities, which were found in differential abundance between the PA and FL fractions (Fig. 2C). The classes *Flavobacteriia*, *Alphaproteobacteria*, and *Gammaproteobacteria* were abundant in the PA and FL fractions, whereas the class *Flavobacteriia*, particularly the family *Cryomorphaceae*, was only present in the PA fraction (Fig. 2). In contrast, the classes *Alphaproteobacteria* (particularly the families *Rhodobacteraceae* and *Pelagibacteraceae*) and *Gammaproteobacteria* (particularly the families *Alteromonadaceae* and *Oceanospirillaceae*) were consistently enriched in the FL fraction only (Fig. 2). Previous studies revealed that the class *Flavobacteriia* was present in various aquatic habitats as a key group of PA bacteria (DeLong *et al.*, 1993; Ortega-Retuerta *et al.*, 2013; Rieck *et‍ ‍al.*, 2015; Suzuki *et al.*, 2017), whereas the class *Alphaproteobacteria* was considered to be a FL type (DeLong *et al.*, 1993; Crespo *et al.*, 2013). Overall, the abundance of the class *Flavobacteriia* was higher (not significant; *P*>0.05) in eelgrass-covering (inside) water than in bare-ground (outside) water (Fig. 2A). In addition, the family *Cryomorphaceae* (*Flavobacteriia*) was found in a PA form from inside water (Fig. 2C), whereas this group was previously identified as a group of bacterioplankton in seagrass-surrounding waters (Ugarelli *et al.*, 2019). Members of *Gammaproteobacteria* have rarely been reported as a primary lineage of FL bacteria (DeLong *et al.*, 1993; Suzuki *et al.*, 2017). The relative abundance of *Gammaproteobacteria* slightly differed between these studies and the present study (Fig. 2A and 2B). This group’s rate discrepancy was attributed more to habitats or methodological differences than to sample processing and technical errors. Yung *et al.* (2016) reported that some groups belonging to *Gammaproteobacteria* switched their lifestyle between PA and FL (Yung *et al.*, 2016), depending on their substrate availability (Grossart, 2010; Li *et al.*, 2021). The class *Gammaproteobacteria* has been shown to adopt a dual-lifestyle strategy (Yung *et al.*, 2016; Li *et al.*, 2021) and, thus, survives and grows not only in the PA state, but also in the FL state (Grossart, 2010; Li *et‍ ‍al.*, 2021). The groups *Cyanobacteria*, *Planctomycetes* [Saprospirae], *Verrucomicrobia*, and *Deltaproteobacteria* were exclusively identified in suspended particles (Fig. 2A and 2B). These groups have been detected in suspended particles in various marine habitats (Crespo *et al.*, 2013; Mohit *et al.*, 2014; Bižić-Ionescu *et al.*, 2015; Rieck *et al.*, 2015; Salazar *et al.*, 2015). The groups *Planctomycetes* and *Verrucomicrobia* are known to be particle degraders (Woebken *et al.*, 2007; Bachmann *et al.*, 2018), which may play an important role in bio-element cycling in a seagrass bed. In addition, although there was a temperature difference of nearly 10°C between the maximum and minimum (Table 1), FL microbial communities were similar to each other (Fig. 2A). Regarding PA fractions, the abundance ratios of major groups, such as *Alphaproteobacteria* and *Gammaproteobacteria*, differed slightly from region to region (Fig. 2A).

**Fig. 3.**
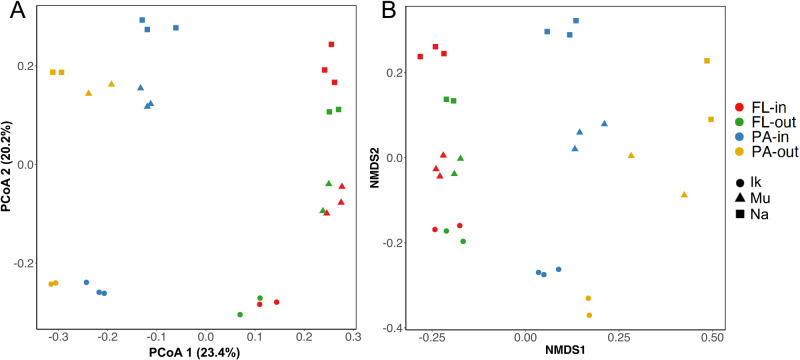
(A) Principal coordinates ana­lysis (PCoA) and (B) nonmetric multidimensional scaling (NMDS) based on unweighted Unifrac distances matrices of microbial communities associated with PA and FL fractions of eelgrass-covering water (inside) and bare-ground water (outside) collected from three *Zostera marina* eelgrass fields. Samples are colored by sample type (PA-in: particle-attached inside; PA-out: particle-attached outside; FL-in: free-living inside; FL-out: free-living outside), with different shapes for sampling areas (Ik: Ikuno-Shima Is.; Mu: Mutsu Bay; Na: Nanao Bay).

**Fig. 4.**
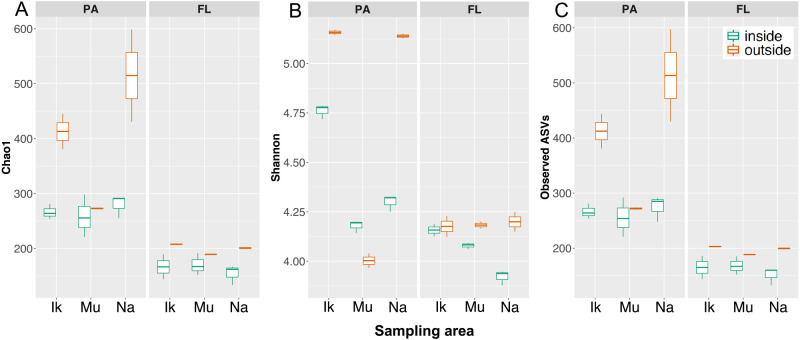
Alpha diversity of test samples. Three alpha diversity metrics, (A) Chao1, (B) the Shannon diversity index, and (C) observed ASVs are shown as boxplots for both the PA (particle-attached) and FL (free-living) fractions of eelgrass-covering water (inside) and bare-ground water (outside) collected from three *Zostera marina* eelgrass fields (Ik: Ikuno-Shima Is.; Mu: Mutsu Bay; Na: Nanao Bay). Alpha diversity was greater in the PA fraction than in the FL fraction (the Wilcoxon rank-sum test: Chao1, *P*<0.001; Shannon, *P*<0.001; observed ASVs, *P*<0.01). The diversity of inside water was lower than that of outside water in both the PA (the Wilcoxon rank-sum test, *P*<0.05) and FL (the Wilcoxon rank-sum test, *P*<0.05) fractions, with the exception of the Shannon index in the PA fraction (the Wilcoxon rank-sum test, Mu: *P*=0.3).

The statistical analysis performed in the present study revealed differences (PERMANOVA; *R^2^*=0.22, *P*<0.001) in the microbial communities in eelgrass-covering waters according to microbial lifestyles (PA and FL; Fig. 3). Previous studies also suggested marked differences in microbial compositions between the PA and FL fractions in various marine habitats (Acinas *et al.*, 1997, 1999; Milici *et al.*, 2017; Bachmann *et al.*, 2018). As shown in Fig. 4, alpha diversity was greater within the PA fraction than within the FL fraction (the Wilcoxon rank-sum test: Chao1, *P*<0.001; Shannon, *P*<0.001; Observed ASVs, *P*<0.01; see also Table S1). These results are consistent with previous findings (Crespo *et al.*, 2013; Ortega-Retuerta *et al.*, 2013; Rieck *et al.*, 2015; Milici *et al.*, 2017).

Diversity was lower in inside water than in outside water in both the PA (the Wilcoxon rank-sum test, *P*<0.05) and FL (the Wilcoxon rank-sum test, *P*<0.05) fractions, with the exception of the Shannon index in the PA fraction (the Wilcoxon rank-sum test, Mu: *P*=0.3; Fig. 4 and Table S1). Similar findings were obtained in a recent study on an eelgrass microbial community (Ettinger *et al.*, 2017; Iqbal *et al.*, 2021, 2022). In contrast to expectations, this result implies that microbial diversity is not necessarily rich in domestic eelgrass beds. Diversity characteristics may have been caused by an increased number of rare taxa in outside seawater (Ettinger *et al.*, 2017). However, no significant differences were observed in alpha diversity among the three sampling areas (Ikuno-Shima Is., Mutsu Bay, and Nanao Bay) for both the PA (Kruskal-Wallis, *P*>0.05) and FL (Kruskal-Wallis, *P*>0.05) fractions, except for the PA fraction of the Shannon index chart (Kruskal-Wallis, *P*<0.05; Fig. 4 and Table S1). Similar results were obtained for the Futtsu eelgrass bed, where alpha diversity was higher within the PA fraction than within the FL fraction (Iqbal *et al.*, 2023). Furthermore, diversity was lower in inside water than in outside water, with only one exception (Iqbal *et al.*, 2021).

Regarding samples of eelgrass-covering water, the PERMANOVA test revealed a significant difference in the microbial composition between the PA microbial community and FL microbial community (PERMANOVA; *R^2^*=0.22, *P*<0.001). In addition, the two-way PERMANOVA test revealed that the PA microbial community varied (two-way PERMANOVA; *R^2^*=0.12, *P*<0.05) with a difference not only in sampling areas (Ikuno-Shima Is., Mutsu Bay, and Nanao Bay), but also in sampling points (inside and outside), whereas the FL microbial community were not markedly affected (two-way PERMANOVA; *R^2^*=0.09, *P*>0.05) by spatial differences (Table 2).

Ward *et al.* (1984) suggested that the resuspension of marine sediments by waves changed suspended particle concentrations in a shallow embayment colonized by seagrasses. In a seagrass vegetation area, since water movement was found to be restricted by the resistance of the seagrass canopy, marine sediments were less likely to roll up than in adjacent unvegetated areas (Orth, 1977; Ward *et al.*, 1984; Gillis *et al.*, 2014). Therefore, the number of particles inside the eelgrass bed became smaller than outside the eelgrass bed (Ward *et al.*, 1984). If microbes that do not normally exist in the water are supplied by sediment roll-up, the microbial diversity of these particles may increase depending on the degree of sediment resuspension. In the present study, the microbial diversity of suspended particles outside (bare-ground) eelgrass colonies markedly exceeded that inside (eelgrass-covering), particularly in the two sea areas of Ikuno-Shima and Nanao (Fig. 4). It is reasonable to speculate that the eelgrass canopy inhibited the sediment surface from rolling up (Orth, 1977; Ward *et al.*, 1984), which eventually reduced microbial recruitment from the seafloor. Regarding the samples from Mutsu, no significant difference was observed in the microbial diversity of particles between bare-ground water and eelgrass-covering water (Fig. 4). We speculate that the effects of sediment roll-up were small, in contrast to the other two eelgrass areas, due to large sediment particles with low organic matter (Iqbal *et al.*, 2021; Table 1). This result implies a negligible microbial supply from the sediment surface to the water layer in the Mutsu eelgrass area. However, even at Mutsu, differences in the PA microbial community between sampling points (in and out) were greater than those in the FL microbial community (Fig. 3). This suggests that the PA microbial community above the eelgrass colony was affected by factors other than sediment roll-up.

A previous study reported that the eelgrass bed had a qualitative impact on the surrounding seawater throughout the year in its life cycle of growth and collapse (Moore, 2004). Hemminga *et al.* (1991) made several important statements on the leakage of nutrients from seagrass beds. Seagrasses release 2% of the carbon fixed in photosynthesis as DOM (Hemminga *et al.*, 1991). Furthermore, some of the eluted nutrients were retained in seagrass beds, while the rest was released into the water layer (Hemminga *et al.*, 1991; Wilkie *et al.*, 2012; Gillis *et al.*, 2014). If the water directly above the eelgrass bed (eelgrass-covering water) contains eluted nutrients, microbial communities that utilize dissolved nutrients may induce changes in the overall microbial community structure. However, as shown in Fig. 4, the diversity of the FL microbial community was similar between eelgrass-covering (inside) water and bare-ground (outside) water in the three eelgrass beds examined. Further evidence to show that the eluted nutrients did not affect the FL microbial community was noted in the PCoA and NMDS maps, on which each other’s groups (FL-in and FL-out) were plotted close together regardless of the presence or absence of eelgrasses (Fig. 3). In contrast, the PA microbial community appeared to differ between the two sampling points (PA-in and PA-out; Fig. 3). Structural differences in the PA microbial community between the inner and outer eelgrass colonies were significant (Table 2). This implies that the PA microbial community was slightly affected by the presence of eelgrass colonies (Table 2 and Fig. 3). Robertson *et al.* (1982) suggested in their laboratory simulations that seagrass-derived dissolved organic carbon (seagrass DOC) is readily converted into bacterial aggregates, which may be ingested by higher-order predators in detritus food webs. A picture of the eelgrass leaf surface indicates the presence of amorphous fluffy substances covering withered leaves (Fig. S2). This fluffy substance is considered to have originated from the conversion of seagrass DOC, and may easily peel off with strong hydrodynamic events and marine herbivore consumption and drift to the surrounding water (Kirkman and Reid, 1979; Hemminga *et al.*, 1991; Gillis *et al.*, 2014: Fig. S2). Around eelgrass colonies, the marine food web is supported by the primary production of eelgrass, unlike the open ocean, which depends on the primary production of phytoplankton. Since organic matter derived from dead leaves is deposited around the eelgrass colony, PA microbes are assumed to mainly utilize sedimentary organic matter derived from eelgrass detritus. On the other hand, the contribution of organic matter directly leaking from the eelgrass leaf surface cannot be ignored.

In conclusion, PA microbes in eelgrass fields are generally formed by sediment roll-up and organic matter leaking from eelgrass leaves (Fig. S3). However, the PA microbial community within the eelgrass bed is less susceptible to sediment roll-up than that outside the eelgrass bed because waves and tidal currents are attenuated by eelgrass vegetation (Orth, 1977; Ward *et al.*, 1984). In addition, the PA microbial community within the eelgrass bed may differ from that outside the eelgrass bed, independent of the sediment roll-up effect, because of the continuous supply of DOC leaking from eelgrass leaves within the eelgrass bed (Kirkman and Reid, 1979). Many sequences of the key groups (*e.g.*, *Planctomycetes* and *Verrucomicrobia*) were found in the PA fraction. These groups are known to be particle degraders and, thus, are expected to be key players in bio-element cycles in eelgrass beds.

## Citation

Iqbal, M. M., Nishimura, M., Sano, M., and Yoshizawa, S. (2023) Particle-attached Microbes in Eelgrass Vegetation Areas Differ in Community Structure Depending on the Distance from the Eelgrass Bed. *Microbes Environ ***38**: ME23013.

https://doi.org/10.1264/jsme2.ME23013

## Supplementary Material

Supplementary Material

## Figures and Tables

**Table 1.** Environmental variables of three collection areas (Ikuno-Shima Is., Mutsu Bay, and Nanao Bay). Test samples were collected from two sampling points (inside and outside).

Sampling area & date	Location coordinates	Sampling point	Depth (m)	Temperature (°C)	Salinity (PSU)*	pH	ORP**	Sediment TOC (mg g^–1^)^†^	Sediment grain size (μm)^†^
Ikuno-Shima Is., Hiroshima June-2015	34°17′52.92″N, 132°55′02.91″E	Inside	0.8	19.8	31.6	8.4	173	9.92±0.82	20.3
	34°17′52.45″N, 132°55′01.40″E	Outside	0.8	20.0	32.4	8.3	196	3.09±0.37	129.3
Mutsu Bay, Aomori Aug-2015	40°51′58.06″N, 140°50′00.04″E	Inside	1.4	26.4	32.1	8.2	166	1.75±0.30	133.8
	40°51′59.31″N, 140°50′00.20″E	Outside	1.4	26.8	32.7	8.3	180	0.90±0.13	215.6
Nanao Bay, Ishikawa July-2015	37°05′10.21″N, 136°52′14.62″E	Inside	1.0	28.2	30.8	8.2	158	3.94±0.31	72.6
	37°05′09.43″N, 136°52′16.16″E	Outside	0.9	28.4	30.6	8.2	77	5.18±0.28	117.8

*) PSU, Practical salinity unit, **) ORP, Oxidation-reduction potential, †) Some data have been published in Iqbal *et al.,* 2021.

**Table 2.** Two-way permutational multivariate ana­lysis of variance (two-way PERMANOVA) test for the significance of unweighted Unifrac distances of microbial communities associated with PA and FL fractions according to listed factors.

Two-way PERMANOVA										
Factors	*df*	PA			Pr(>F)	*df*	FL			Pr(>F)
		MS	F.Model	*R^2^*			MS	F.Model	*R^2^*	
Sampling area	2	0.69926	7.0007	0.46715	0.0001 ***	2	0.54421	12.1009	0.63387	0.0001 ***
Sampling point	1	0.31944	3.1981	0.1067	0.0026 **	1	0.11156	2.4805	0.06497	0.0532
Sampling area: Sampling point	2	0.1884	1.8861	0.12586	0.0238 *	2	0.07867	1.7493	0.09163	0.1069
Signif. Codes: ‘***’ 0.001 ‘**’ 0.01 ‘*’ 0.05 ‘.’ 0.1 ‘ ’ 1										
