# Supplementary material for: Particle-attached Microbes in Eelgrass Vegetation Areas Differ in Community Structure Depending on the Distance from the Eelgrass Bed
Source: Microbes Environ. 2023 Sep 2;38(3):ME23013. doi: 10.1264/jsme2.ME23013 (PMC10522840; doi:10.1264/jsme2.ME23013)
Supplement: Supplementary file 1 — Supplementary Material [file 38_23013_s1.pdf]

Supplementary data

Table S1. Statistics of Illumina sequencing data and alpha diversity (Chao1, Shannon, and observed ASVs) values of each sample collected from three *Z. marina* eelgrass fields (Ikuno-Shima Is., Mutsu Bay, and Nanao Bay) in Japan.

Sampling area	Sampling point	Fraction	replicate	Illumina sequencing data						Alpha diversity	
				Before rarefied				After rarefied			
				Raw sequences	Non-chimeric	Filtered sequences	Number of ASVs	Filtered sequences	Number of ASVs (Observed)	Chao1	Shannon
Ikuno-Shima Is.	Inside	PA	R1	121956	77482	36070	264	29885	264	264.0	4.72
			R2	104859	66061	29885	254	29885	254	254.0	4.79
			R3	88636	67722	30465	281	29885	281	281.0	4.78
		FL	R1	150925	110393	110087	186	29885	186	189.3	4.19
			R2	74073	51368	51211	144	29885	144	144.0	4.13
	Outside	PA	R1	97324	72681	36699	381	29885	381	381.0	5.14
			R2	136676	103066	53513	445	29885	444	445.3	5.17
		FL	R1	104713	88896	88661	208	29885	204	206.6	4.23
			R2	102809	83817	83576	204	29885	202	209.0	4.12
Mutsu Bay	Inside	PA	R1	101383	78130	75307	298	29885	292	298.0	4.20
			R2	75138	58926	56636	222	29885	221	221.3	4.14
			R3	89950	69842	67499	255	29885	254	255.7	4.19
		FL	R1	122059	95622	95265	188	29885	185	192.0	4.08
			R2	96039	81349	81034	168	29885	167	167.3	4.09
			R3	91412	76922	76702	152	29885	152	152.0	4.06
	Outside	PA	R1	75518	56163	48892	274	29885	274	274.4	3.97
			R2	94537	74372	70890	272	29885	270	271.4	4.04
		FL	R1	106249	89419	88996	190	29885	188	188.8	4.17
			R2	106818	87242	86785	191	29885	189	190.0	4.20

Cont. Table S1

Sampling area	Sampling point	Fraction	replicate	Illumina sequencing data						Alpha diversity	
				Before rarefied				After rarefied			
				Raw sequences	Non-chimeric	Filtered sequences	Number of ASVs	Filtered sequences	Number of ASVs (Observed)	Chao1	Shannon
Nanao Bay	Inside	PA	R1	114085	92104	80760	292	29885	291	292.2	4.32
			R2	111066	90736	79440	287	29885	285	291.0	4.33
			R3	97928	80645	70942	250	29885	248	255.5	4.25
		FL	R1	119817	98939	98796	163	29885	160	162.1	3.94
			R2	124534	106620	106549	161	29885	160	167.0	3.95
			R3	99633	81758	81706	134	29885	133	133.5	3.88
	Outside	PA	R1	82265	60907	52498	430	29885	430	430.9	5.15
			R2	114027	92439	82173	601	29885	597	598.8	5.13
		FL	R1	115538	98336	98102	198	29885	198	199.1	4.15
			R2	116914	98249	98050	204	29885	201	202.7	4.25

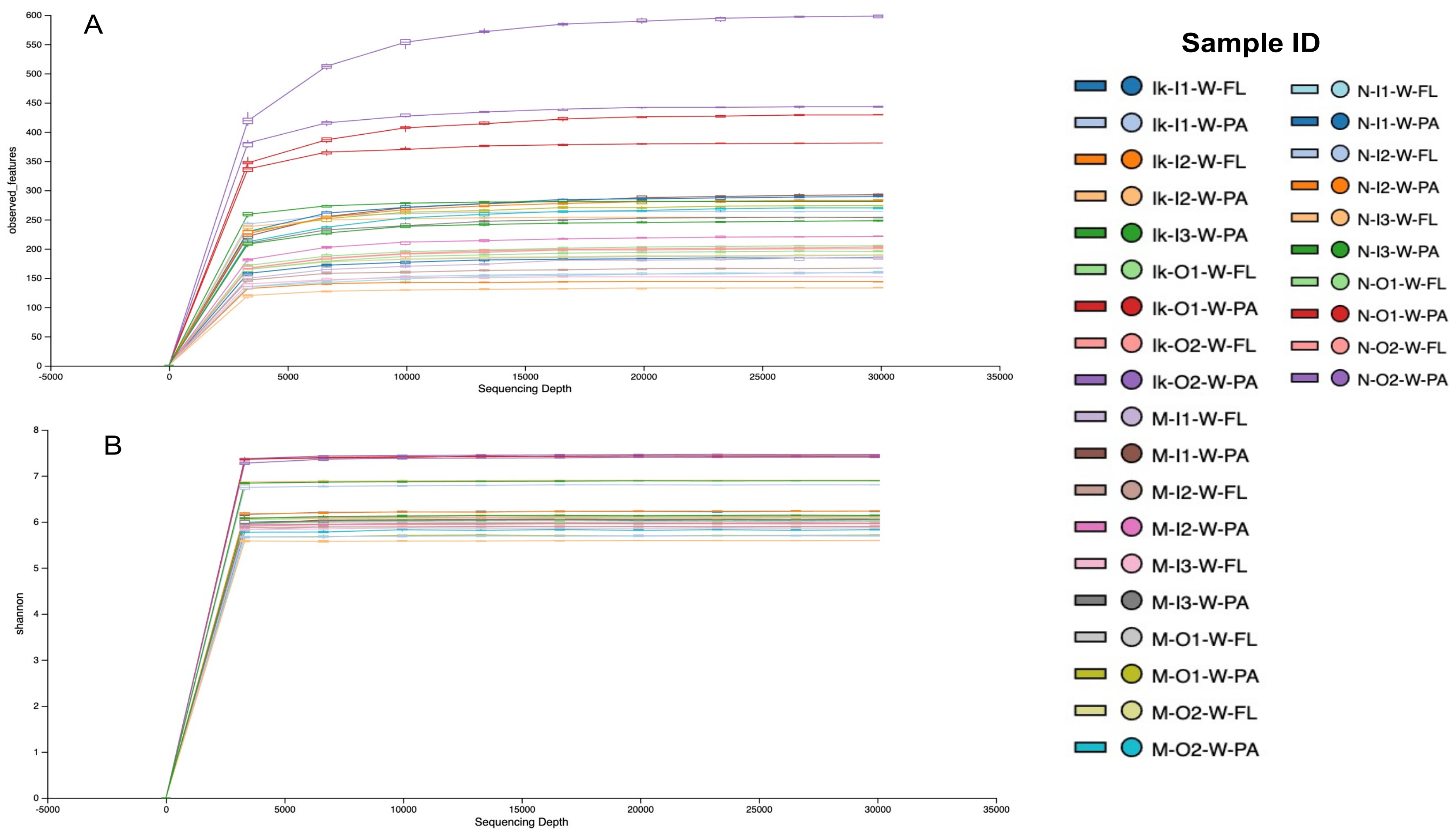

Fig. S1. Rarefaction curves were generated based on (A) observed amplicon sequence variants (ASVs) and (B) Shannon diversity index measure for each sample. Where the sample IDs' expressing; Ik: Ikuno-Shima Is.; M: Mutsu Bay; N: Nanao Bay; (I1, I2, I3): inside eelgrass bed; (O1, O2): outside eelgrass bed; W: seawater sample; PA: particle-associated; FL: free-living.

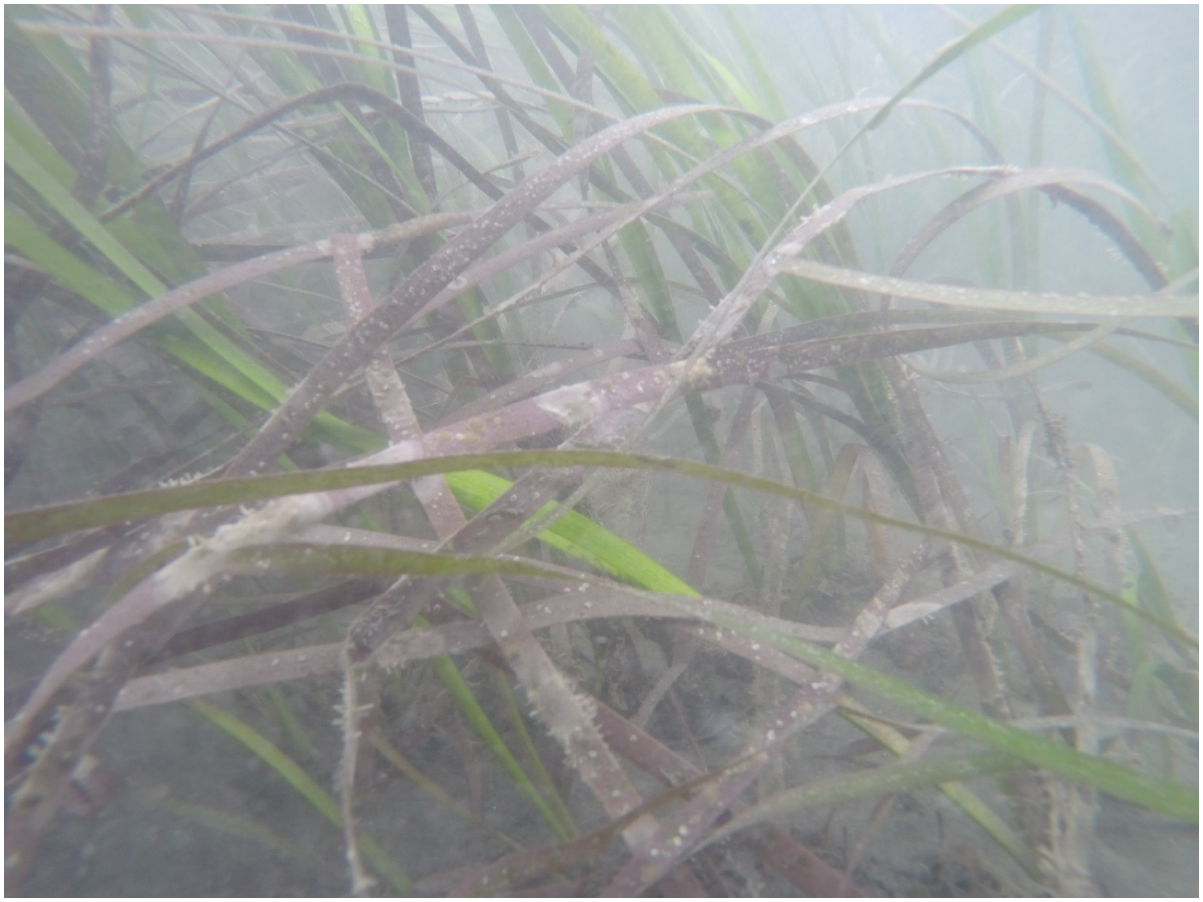

Fig. S2. The surface of eelgrass leaves, especially withered leaves, is covered with amorphous fluffy substances. They are likely to peel off easily when washed by waves. This photo was taken at Futtsu eelgrass bed (35°18′56.91″N,139°47′42.03″E), Chiba prefecture, Japan in the summer (July 05, 2016, Iqbal *et al*., 2021).

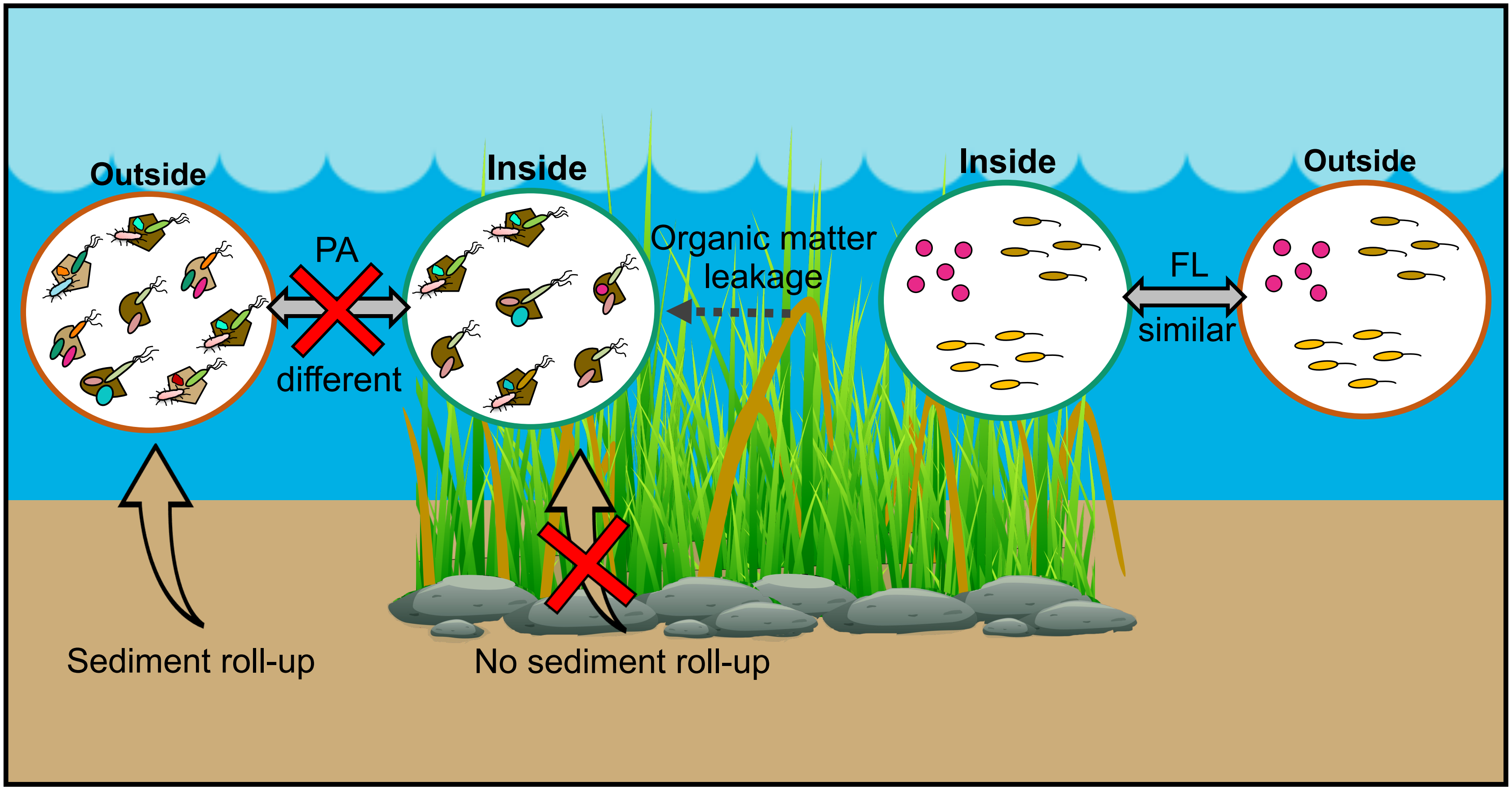

Fig. S3. Conceptual diagram illustrating how PA-microbial communities are affected by the eelgrass colony. PA: particle-attached; FL: free-living.
